# Lessons from the epidemiological investigation efforts during the COVID-19 epidemic in Israel: Liron Inchi

**DOI:** 10.1093/eurpub/ckac131.086

**Published:** 2022-10-25

**Authors:** L Inchi, S Bord, B Madjar

**Affiliations:** 1 Health Systems Management, The Max Stern Yezreel Valley College, Yezreel Valley, Israel; 2 Haifa District, Ministry of Health, Jerusalem, Israel

## Abstract

**Background:**

The COVID-19 pandemic caused a crisis in the Israeli healthcare system. The wave-like morbidity created an overload of epidemiological investigations, which led to delays and less than successful efforts to prevent infection. For this reason, Israel decided to mobilize the military for this purpose, creating a forced cooperation between the Ministry of Health (MOH) and the IDF, an expert in dealing with crises.

**Aim:**

To examine the implications of the forced encounter between the IDF and the healthcare system, including both tensions and cooperation efforts.

**Methods:**

Twenty in-depth interviews were carried out with MOH and IDF personnel in different roles at various levels towards the end of the second pandemic wave in Israel.

**Findings:**

The findings present a dual picture of cooperation and mutual respect, side by side with contradictions and conflicts. The feeling that the IDF came in to ‘save the day’ placed the healthcare people in an inferior position. Clearly, there was no explicit plan for division of authority. It was clear to the healthcare staff that they have the authority as the professionals, and to the IDF people that they have it as the ‘saviors’ brought in for this purpose. The healthcare people did see the potential of the military force mobilized for this purpose, but felt they were asked for their opinion only initially, when the military personnel had to study the system. As soon as they became familiar with it, they were no longer asked for advice, and control was given to the IDF. The findings also show that the MOH’s qualitative professional approach often clashed with the IDF’s action-based approach.

**Conclusions:**

The military was mobilized as a crisis expert in order to assist the healthcare system in managing said crisis, but in fact this assistance had many side effects. Managing expectations, division of authority and open, sharing communication may have been useful in preventing the conflicts and managing them.

**Key messages:**

• It is important to be aware of the a-symmetrical power relations of the military-healthcare system interface in order to create effective work and true, coherent cooperation.

• Management of expectations, organized division of authority and open, sharing communication may have assisted in preventing the conflicts raised and managing them.

